# Supplementary material for: A prospective evaluation of Johnson & Johnson COVID-19 vaccine on glycemic biomarkers in type 2 diabetes mellitus in Ethiopia
Source: PLoS One. 2026 Feb 4;21(2):e0340457. doi: 10.1371/journal.pone.0340457 (PMC12871954; doi:10.1371/journal.pone.0340457)
Supplement: Attachment — Submitted filename: Response to Reviewers.docx [file pone.0340457.s003.docx]

**Response to Reviewers**

**Manuscript ID:** PONE-D-25-35535
**Title:** *A Prospective Evaluation of Johnson & Johnson COVID-19 Vaccine on Glycemic Biomarkers in Type 2 Diabetes Mellitus in Ethiopia*

Dear Dr. Hariyanto,
We sincerely thank you, the Academic Editor, and the reviewers for your valuable time and constructive feedback on our manuscript. We have carefully revised the paper in line with all the points raised. Below, we provide a detailed, point-by-point response. Reviewer comments are restated in *italics*, followed by our responses and corresponding changes in the manuscript.

**Journal Requirements**

**1. Style requirements and file naming**
*Comment:* Please ensure manuscript follows PLOS ONE style templates.
**Response:** We reformatted the manuscript according to PLOS ONE’s formatting requirements. File names for resubmission now follow the required conventions:

- “Response to Reviewers”
- “Revised Manuscript with highlight Changes”
- “Manuscript”

**2. Funding information mismatch**
*Comment:* The ‘Funding Information’ and ‘Financial Disclosure’ sections do not match.
**Response:** We corrected this discrepancy. The Funding section now clearly states:
“There is no funding or grant received for this study.”

**3. Role of funders**
*Comment:* Please clarify the role of funders.
**Response:**
“There is no funder for this study and therefore, no roles.”

**4. Data availability policy**
*Comment:* All PLOS journals require underlying data to be freely available.
**Response:**
“All relevant data within the manuscript were uploaded with the title of supplementary files to be freely accessible.”

**5. Citing suggested studies**
*Comment:* Reviewers recommended citing additional work.
**Response:** We incorporated the following references in the study:

- Marfella R et al., *Nat Commun* 2022.
- Sardu C et al., *J Clin Med* 2022.
- Marfella R et al., *Diabetes Obes Metab* 2022.

**Reviewer #1**

**Major Comments**

1. *Insufficient prior research on glycemic control and vaccine response.*
   **Response:** We revised the Introduction and Discussion to integrate the suggested studies and explain how poor glycemic control may impact vaccine immunogenicity and outcomes.
2. *Clarification of HbA1c trends.*
   **Response:** We rephrased the Results and Discussion to state “elevated HbA1c levels were observed” rather than “sustained increase,” and emphasized potential influence of unmeasured confounders despite the GEE adjustments.
3. *Control group assignment bias.*
   **Response:** We acknowledged the non-randomized nature of the study and discussed potential bias due to self-selection. Additionally, we included the need to conduct sensitivity analysis using Propensity Score Matching (PSM) to adjust for baseline differences as a limitation of the study.
4. *Absence of immunological markers.*
   **Response:** We explicitly acknowledged this limitation in the Discussion and clarified that mechanistic interpretations remain speculative.
5. *Terminology: “improved” vs. “increased.”*
   **Response:** We revised all instances of “improved” to “elevated” or “increased” when describing RBS and HbA1c.

**Minor Comments**

- *Effect size clarification:* We added a discussion on the clinical significance of HbA1c changes.
- *Figures:* All figures are now embedded in the main text and also available as supplementary files.
- *Writing quality:* We edited the Results section for clarity and fluency.

**Reviewer #2**

**Major Comments**

1. *Contextualization to other settings.*
   **Response:** We expanded the Discussion to compare findings in Ethiopia with potential implications for high-income countries, where healthcare infrastructure and vaccination strategies differ.
2. *Limitation: RBS vs. continuous glucose monitoring.*
   **Response:** We acknowledged that RBS is a less robust marker than continuous or flash glucose monitoring, but explained this was due to resource limitations in our setting.
3. *Expanded clinical implications.*
   **Response:** The Conclusion now advises that clinicians may consider closer glycemic monitoring post-vaccination in younger and female T2DM patients, particularly those with poor baseline glycemic control.

**Minor Comments**

1. *Table 2 formatting:* Reformatted for clarity with improved column separation.
2. *Acronyms:* Revised throughout the manuscript for consistency.

**Conclusion**

We believe that the revised manuscript has significantly improved in scientific rigor, contextualization, and clarity following the reviewers’ helpful comments. We are grateful for the opportunity to resubmit and look forward to your further consideration.

Sincerely,
Chala Kenenisa Edae
