# Supplementary material for: A prospective evaluation of Johnson & Johnson COVID-19 vaccine on glycemic biomarkers in type 2 diabetes mellitus in Ethiopia
Source: PLoS One. 2026 Feb 4;21(2):e0340457. doi: 10.1371/journal.pone.0340457 (PMC12871954; doi:10.1371/journal.pone.0340457)
Supplement: Attachment — Submitted filename: Response to Review Comments.docx [file pone.0340457.s004.docx]

**Response to Review Comments**

We thank the reviewers for their careful review and constructive feedback, which have helped improve the clarity and quality of the manuscript. We address the comments below.

**Reviewer #1**

**Comment:**
The manuscript has been further improved and refined for clarity and overall quality. Overall, the scientific message is acceptable.

**Response:**
We thank the reviewer for the positive assessment of the manuscript and for acknowledging the improvements made. We are pleased that the scientific message is considered sound.

**Reviewer #3 – Minor Comments**

**Comment:**
Condense lines 1–3 of the Introduction to avoid repetition of global DM prevalence.

**Response:**
The Introduction has been revised to condense lines 1–3 and remove repetitive statements on global diabetes mellitus prevalence.

**Comment:**
Define all abbreviations (e.g., GEE, PSM) at first mention.

**Response:**
All abbreviations, including GEE (Generalized Estimating Equations) and PSM (Propensity Score Matching), are now defined at first mention in the text.

**Comment:**
Correct the typo “uninfected group” to “unvaccinated group.”

**Response:**
This typographical error has been corrected throughout the manuscript.

**Comment:**
Consider adding 95% confidence interval ribbons or error bars to trend figures.

**Response:**
Trend figures have been updated to include 95% confidence intervals where appropriate to improve clarity and interpretability

We appreciate the reviewers’ suggestions, which have strengthened the manuscript.
